# Robot-assisted congenital diaphragmatic hernia repair in adults: A case series

**DOI:** 10.1097/MD.0000000000039918

**Published:** 2024-10-25

**Authors:** Yu-Jen Huang, Yue-Lin Fang

**Affiliations:** a Department of General Surgery, Shin Kong Wu Ho-Su Memorial Hospital, Taipei, Taiwan.

**Keywords:** adult patients, case series, congenital diaphragmatic hernia, robot-assisted surgery

## Abstract

**Rationale:**

Congenital diaphragmatic hernia (CDH) is a rare condition predominantly affecting neonates, with only a few cases remaining undetected until adulthood. Surgical repair is the primary treatment approach for adults with confirmed CDH. Traditionally, these procedures include laparotomy, thoracotomy, and minimally invasive techniques such as thoracoscopy and laparoscopy. However, only a few cases of robotic diaphragmatic hernia repair have been reported in recent decades.

**Patient concerns:**

The patients, aged 31 and 71 years, presented with atypical symptoms of chest tightness and fever. Imaging studies revealed a left-sided Bochdalek CDH in 1 patient and a right-sided Morgagni CDH in the other.

**Diagnoses:**

The patients were diagnosed as CDH in adult with different symptoms.

**Interventions:**

Both patients received robot-assisted diaphragmatic hernia repair at our institution.

**Outcomes:**

The patients received robotic-assisted diaphragmatic hernia repair with acceptable surgery outcome and safety. There was no complication or recurrence.

**Lessons:**

This case series indicates that the robotic transabdominal approach for CDH repair in adults can be an optimal minimally invasive approach for selected patients, demonstrating adequate surgical safety and favorable outcomes.

## 1. Introduction

Congenital diaphragmatic hernias (CDHs) are believed to result from the incomplete fusion of the pleuroperitoneal membranes. When the muscular components of the diaphragm develop abnormally, CDHs occur, resulting in the herniation of abdominal contents into the thoracic cavity.^[1]^

The overall incidence of CDH is estimated at 1:2000 to 1:5000 live births.^[2,3]^ Left-sided defects are more common (approximately 80–85%), followed by right-sided defects (15%) and bilateral defects (<5%).^[4]^ Moreover, CDHs, particularly posterolateral left-sided hernias, typically present with symptoms and are identified during the neonatal period, whereas anterior defects may remain undetected until adulthood.^[5,6]^

In adults, the symptoms of CDH can vary widely, from pulmonary or abdominal complaints to asymptomatic cases.^[7,8]^ However, incarceration of intrathoracic abdominal structures can lead to bowel strangulation, necessitating emergency surgical intervention.

Surgical intervention remains the primary strategy for the treatment of CDHs in adults.^[9]^ Minimally invasive techniques have become increasingly prevalent in abdominal surgeries and are now being applied to hernia repair in adults, including laparoscopy, thoracoscopy, and robotic surgery.^[10]^

Robot-assisted surgery has rapidly emerged and is now used for diaphragmatic hernia repairs.^[11]^ With the maturation of the robotic platform, the transabdominal and transthoracic robotic approaches seem to be effective treatment options, enhancing surgical outcomes by offering surgeons better visualization, precision, and dexterity in a limited space.^[11]^ Only a few cases of diaphragmatic hernia repair using robotic platforms have been reported, with minimal associated morbidity and complications.^[12]^

We conducted a retrospective analysis of 2 case series of CDH repair in adults using a robotic platform as a minimally invasive approach at our institution. These cases involved different types of CDHs and rare clinical presentations. Both patients underwent robot-assisted transabdominal hernia repair, which demonstrated acceptable safety and outcomes.

## 2. Case presentation

### 2.1. Case 1

A 31-year-old woman with congenital intellectual disability and a history of epilepsy under medication control presented with anterior chest tightness that had persisted for approximately 3 weeks, along with long-term constipation managed with laxatives. There were no associated symptoms such as shortness of breath, dyspnea on exertion, palpitations, chest pain, or dizziness during this period. However, she experienced an acute onset of severe and intolerable chest pain. After visiting an outpatient clinic, she was transferred to our emergency department for further evaluation. Upon admission, her vital signs were within normal ranges. According to her family, she had experienced intermittent right upper quadrant abdominal colic pain over the past year. This pain was not accompanied by symptoms such as nausea, vomiting, tarry or bloody stools, or diarrhea during the year before presentation. The patient did not seek medical attention earlier because the pain was tolerable and intermittent. The patient’s family said the patient had no history of abdominal or thoracic trauma.

**Figure 1.**
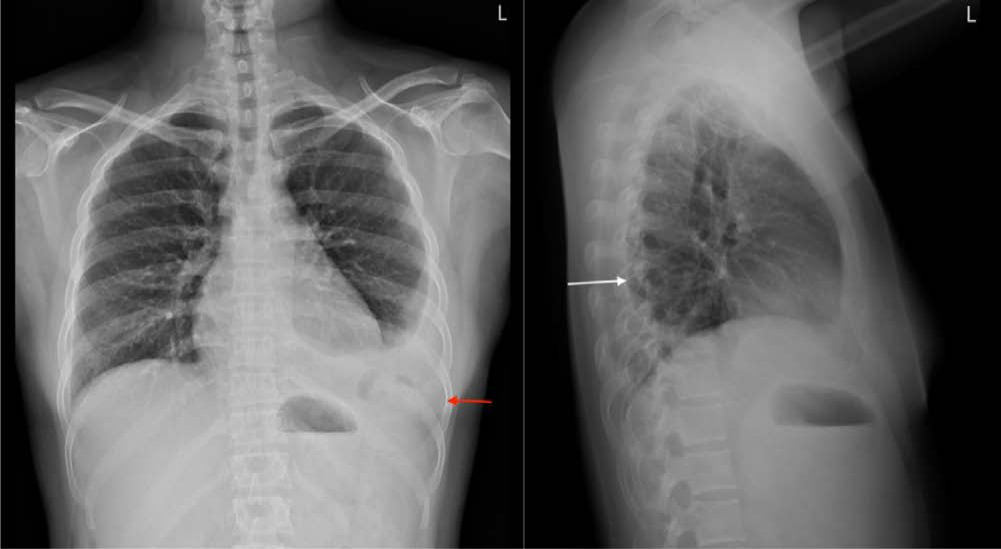
Plain chest radiography for left-sided Bochdalek hernia. Left-sided pleural effusion with suspected bowel contents above the left-sided diaphragm on chest radiography posterior-anterior view (red arrow). Chest radiography lateral view showed some gas and fluid-filled content presented in the pleural cavity, and a left-sided diaphragmatic hernia was suspected (white arrow).

**Figure 2.**
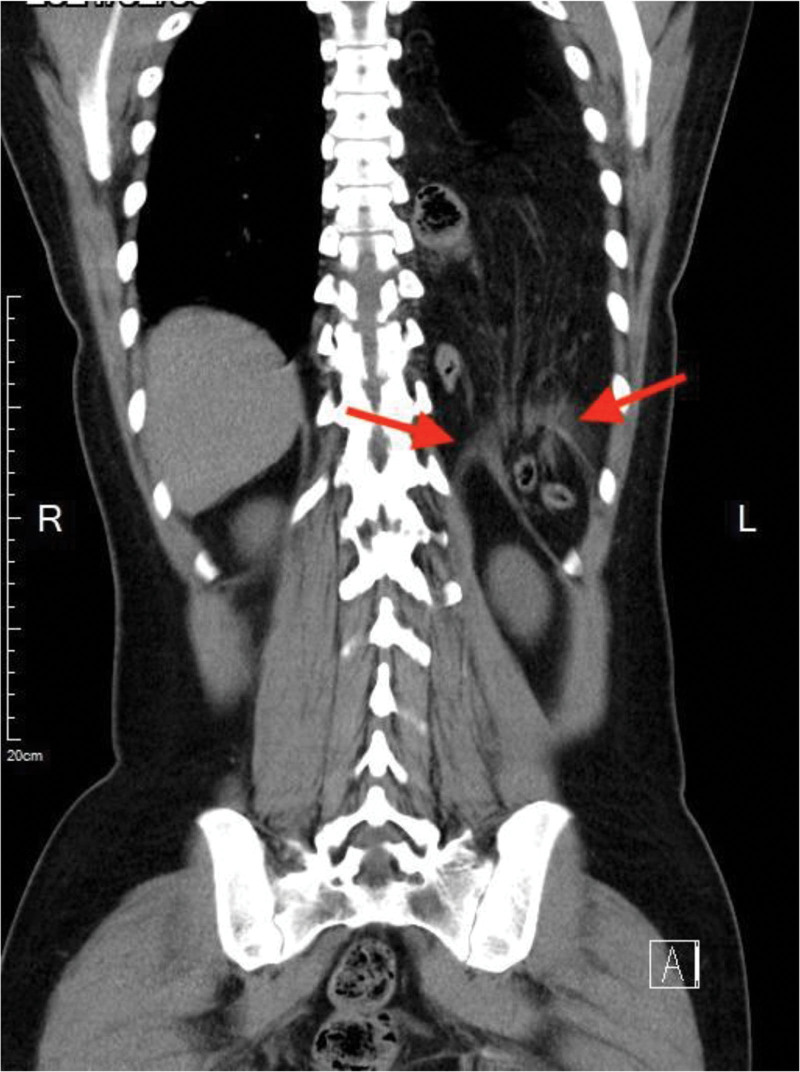
A left-sided Bochdalek hernia in Computed tomography scan. A left-sided Bochdalek hernia defect (red arrow) containing intra-abdominal contents (bowels and omental tissues) is noted on a thoracoabdominal computed tomography scan without contrast (coronal view).

In our emergency department, abdominal palpation revealed no tenderness or peritoneal signs, and the chest examination demonstrated no abnormality finding or bowel sound noticed during auscultation. Laboratory tests showed a mild elevation in white blood cell count (11,400/µL) with neutrophils comprising 87%, a normal troponin-I level (<0.0023 ng/mL), and a normal C-reactive protein level (0.40 mg/dL). Electrocardiography revealed sinus tachycardia. Plain chest radiography, including both posterior-anterior and lateral views, revealed left-sided pleural effusion with suspected bowel gas above the level of the left diaphragm (Fig. 1). Computed tomography (CT) from the chest to the pelvis confirmed a left-sided Bochdalek hernia with herniation of the transverse colon and associated omental tissue into the thoracic cavity (Fig. 2). There was no evidence of bowel obstruction or strangulation. The patient was discharged after achieving symptom relief. During a follow-up visit at the outpatient department, an upper gastrointestinal series was performed, which showed organoaxial rotation of the stomach without complete volvulus.

Robot-assisted diaphragmatic hernia repair using the transabdominal approach was planned and performed by a well-trained surgeon using a Da Vinci Xi Robotic Platform (Intuitive Surgical Inc., Sunnyvale, CA). The patient was positioned supine in the reverse Trendelenburg position (30°). Pneumoperitoneum was created using a Veress needle, and 4 trocars were inserted: 1 subumbilically, 1 in the right mid-clavicular line, 1 in the left mid-clavicular line, and 1 in the right anterior axillary line.

**Figure 3.**
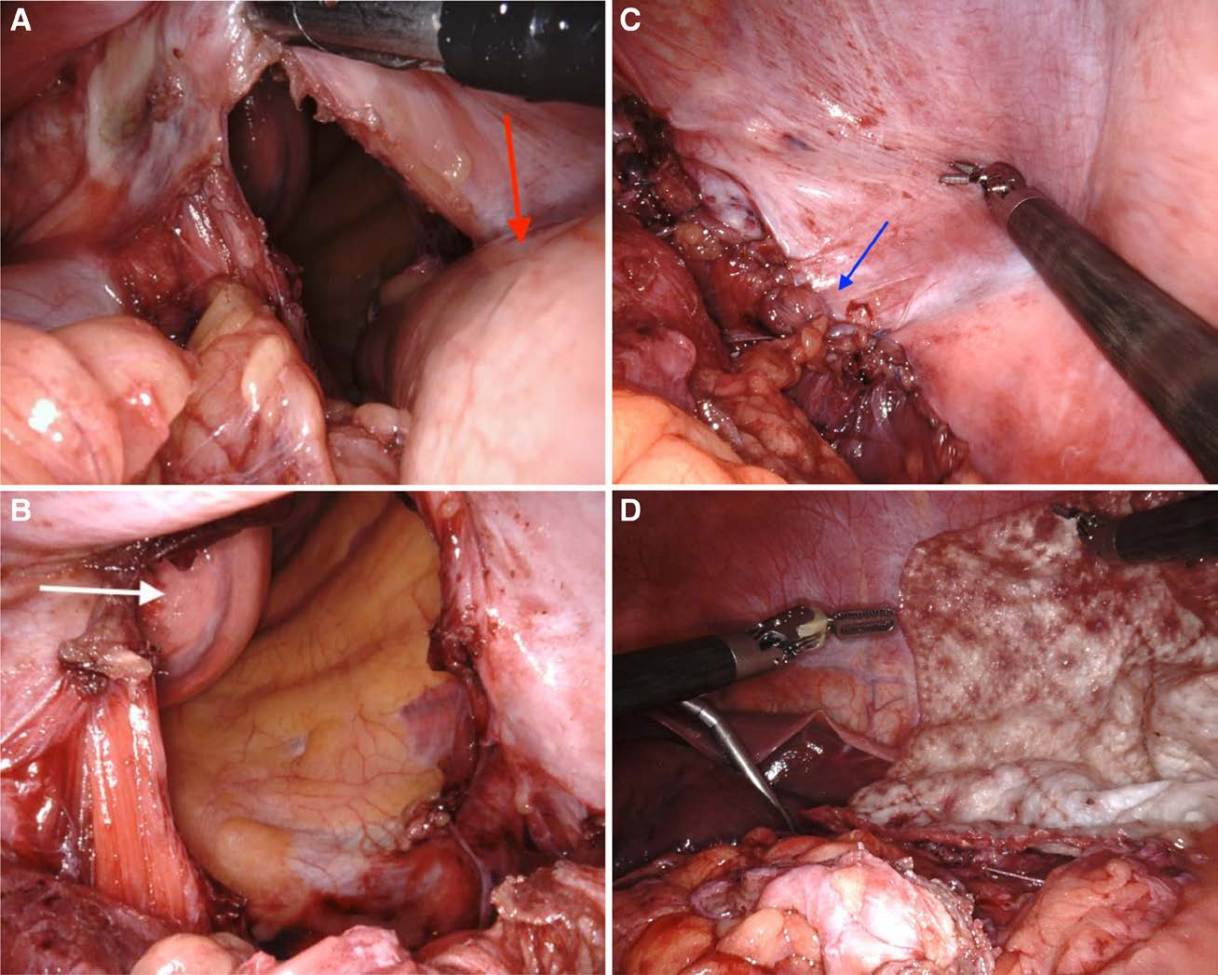
Operative pictures of left-sided Bochdalek hernia repair. (A) Left-sided Bochdalek hernia defect with partial transverse colon (red arrow) incarceration in the left pleural cavity. (B) A large diaphragmatic defect is seen in the left posterior aspect of the diaphragm, with clear visualization of the left lower lung lobe (white arrow). (C) Direct closure of the left-sided Bochdalek hernia with a 2-0 nonabsorbable V-Loc™. (D) Reinforcement with a Biodesign® Hernia Graft (Cook Biotech Inc., West Lafayette, IN).

The incarcerated transverse colon and accompanying omental fat were successfully reduced. The bowel showed no signs of ischemia or poor perfusion, although there was mild swelling following reduction. A 12 × 8 cm diaphragmatic defect was identified in the posterolateral part of the left-sided diaphragm (Fig. 3A), and left lower lung atrophy was noted (Fig. 3B). The hernia repair involved primary closure of the defect using 2-0 nonabsorbable V-Loc™ sutures in a continuous fashion, reinforced with a 22 × 13 cm Biodesign® Hernia Graft (Cook Biotech Inc., West Lafayette, IN) (Fig. 3C and D). A chest surgeon placed a left-sided pigtail drain to decompress the pneumothorax. The estimated blood loss was minimal, and the duration of surgery was 306 minutes. The patient resumed dietary intake on postoperative day 4 and was discharged on postoperative day 8. A follow-up CT performed 6 months later showed no recurrence of the diaphragmatic hernia, with local fibrotic and granulation tissue formation.

### 2.2. Case 2

**Figure 4.**
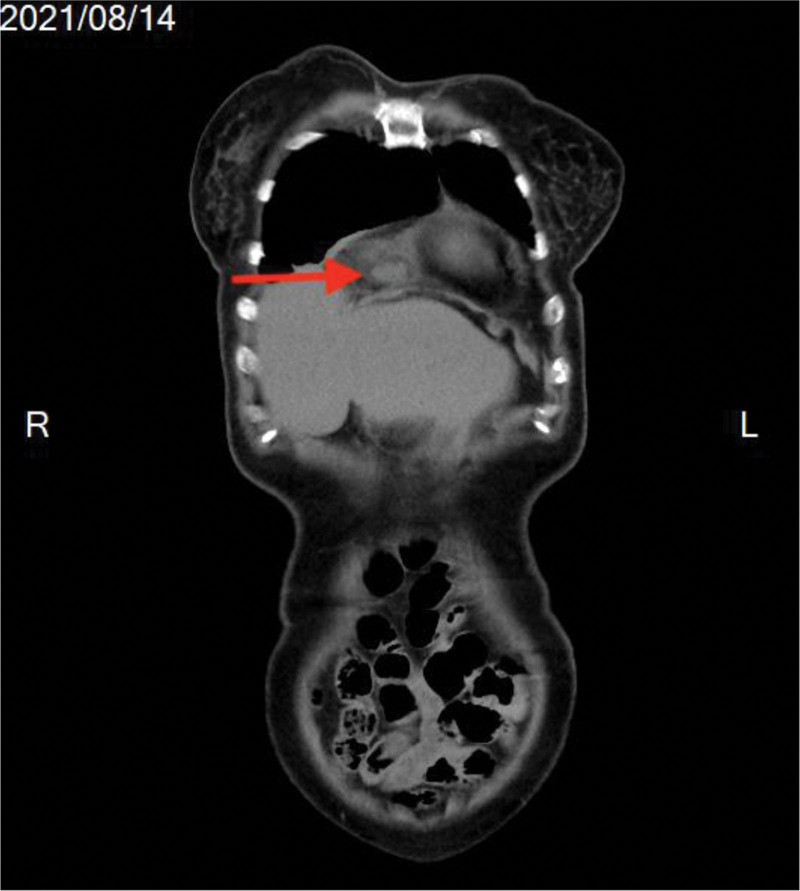
Morgagni hernia showed on the CT scan. A possible incarcerated Morgagni hernia with local omental changes and a hematoma in the sac (red arrow) are seen on the thoracoabdominal computed tomography scan without contrast (coronal view).

**Figure 5.**
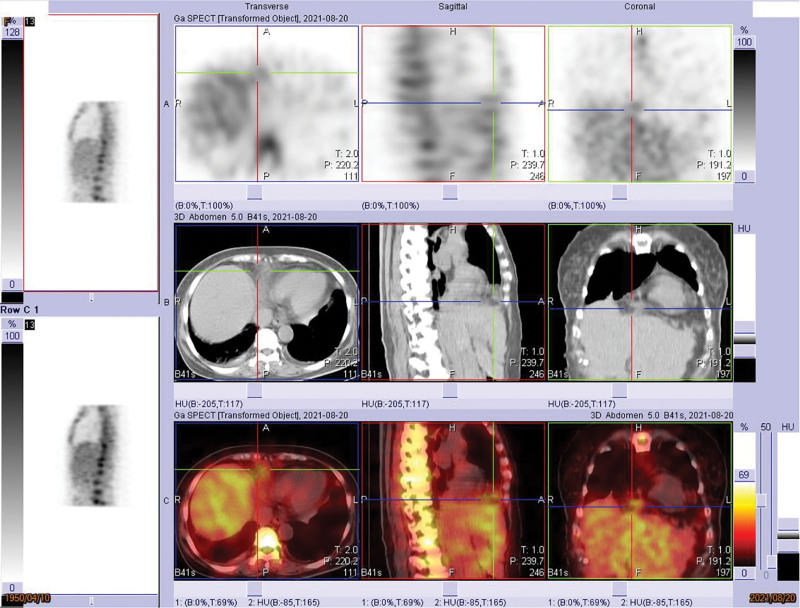
Gallium-67 scan showed inflammatory lesion in the Morgagni hernia. Gallium (Ga-67) inflammation scan showed mildly increased gallium uptake in the soft tissue density lesion in the right anteroinferior mediastinal pericardial space (Morgagni hernia). The scintigraphy findings suggest a possible mild inflammatory lesion in the Morgagni hernia sac.

A 72-year-old woman with a history of systemic diseases, including hypertension, diabetes mellitus, asthma, and dementia, presented to our emergency department with a fever reaching up to 40 °C for 1 day. According to her records, she had received a COVID-19 vaccination approximately 1 month prior and reported no relevant travel history, occupational exposures, contact history, or cluster information of note. She experienced a mild headache, dry cough, and drowsiness but denied any upper respiratory symptoms, abdominal discomfort, or urinary tract infection. On arrival at our emergency department, her temperature was 38 °C. Other vital signs, such as blood pressure, respiratory rate, and heart rate, were within normal ranges. Physical examination of the abdomen revealed no tenderness or peritoneal signs. Mild crackles were noted in the right lower lung field upon auscultation, without bowel sounds. Laboratory examination showed a normal white blood cell count (8900/µL) with neutrophil predominance (81.4%), hyponatremia (133 mmol/L), and an elevated C-reactive protein level (6.51 mg/dL). The first coronavirus PCR test was negative. Computed tomography (CT) from the chest to the pelvis revealed an incarcerated Morgagni hernia with local omental changes and a hematoma in the hernia sac (Fig. 4), complicated by a small right pleural effusion. The patient’s family reported no history of abdominal or chest injury. The patient was admitted to the ward for antibiotic treatment under the care of an infectious disease specialist. A second coronavirus PCR was also negative. Despite improvement in associated symptoms such as dry cough and drowsiness, fever was intermittently observed. Subsequent evaluations revealed nonspecific findings. A gallium scan showed mildly increased gallium uptake in a lesion with soft tissue density in the right anteroinferior mediastinal pericardial space (consistent with the Morgagni hernia). Scintigraphy further revealed a mild inflammatory lesion in the Morgagni hernia sac (Fig. 5).

**Figure 6.**
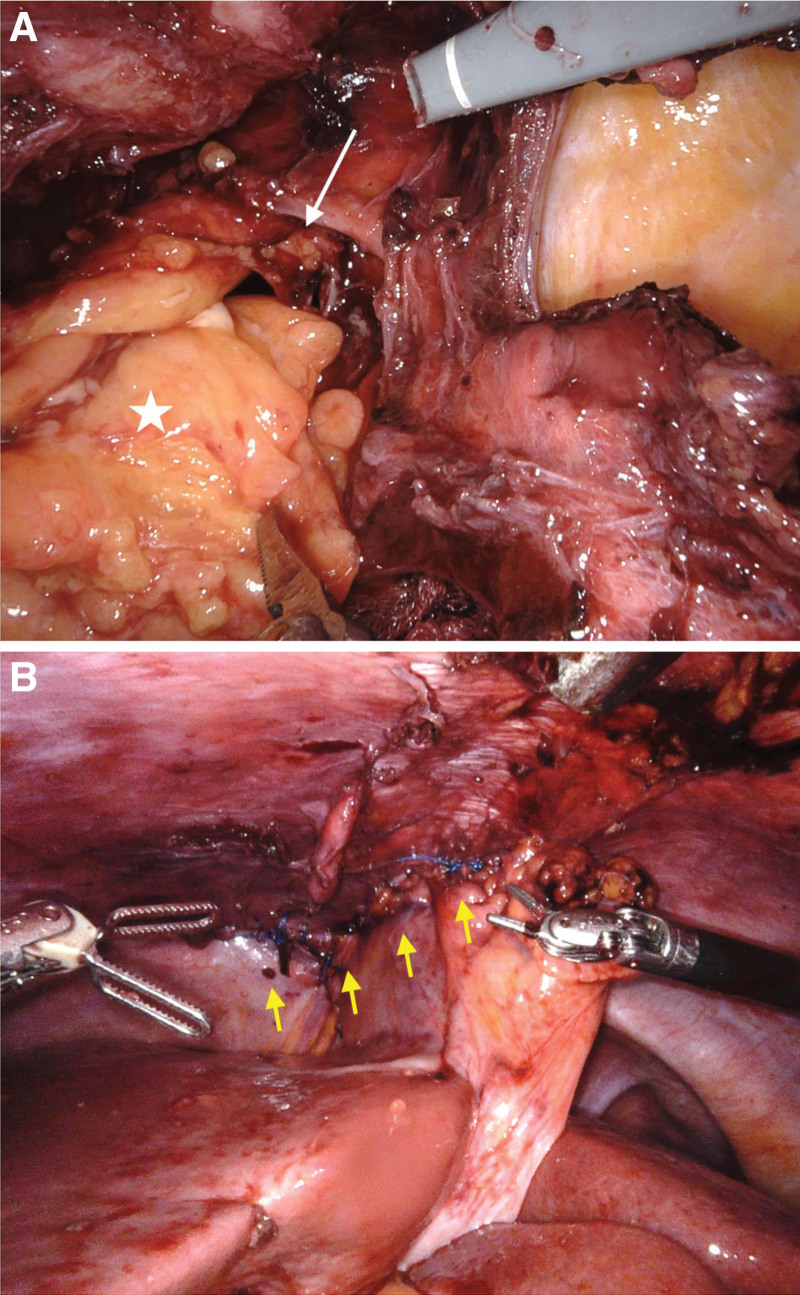
Operative pictures of Morgagni hernia repair. (A) Morgagni hernia (white arrow) with incarcerated omentum and pus (white star). (B) We performed direct closure of the diaphragmatic defect using 2-0 nonabsorbable V-Loc™.

Transabdominal robot-assisted CDH repair was performed with the patient in the supine position and reverse Trendelenburg position (30°). The surgery was conducted using a Da Vinci Xi Robotic Platform (Intuitive Surgical Inc., Sunnyvale, CA) by a well-trained surgeon. Pneumoperitoneum and port placement were achieved using the same techniques as described for Case 1. A diaphragmatic defect measuring 5 × 6 cm was identified at the centro-anterior aspect of the right diaphragm near the falciform ligament. The incarcerated omental fat was reduced from the hernial sac (Fig. 6A). The hernia repair was completed with primary closure of the defect using 2-0 nonabsorbable V-Loc™ sutures in a continuous fashion (Fig. 6B), without additional reinforcement. A chest surgeon performed right-sided pigtail drain insertion to decompress the right-sided pneumothorax. The estimated blood loss was minimal, and the operative time was 287 minutes. The patient received postoperative care in the intensive surgical unit. The fever subsided immediately after surgery. The patient recovered smoothly, was extubated on postoperative day 1, and resumed oral intake on postoperative day 2. On the day following endotracheal tube removal, the patient was transferred to the general ward. The right pigtail drain was removed on postoperative day 6, and the patient was discharged the following day.

A follow-up CT performed 5 months later showed no recurrence of the diaphragmatic hernia, with only local adhesions, granulation tissue, and associated right lower lateral chest wall scar tissue.

## 3. Discussion

We present 2 cases of robot-assisted CDH repair using the transabdominal approach in adults. One patient presented with chest tightness, while the other exhibited the atypical symptom of isolated fever. Both patients underwent robot-assisted surgery with satisfactory postoperative outcomes. The application of robotic platforms for CDH repair in adults remains rare.

CDH presenting in adulthood poses a challenge to clinicians because most patients are either asymptomatic or exhibit vague symptoms, necessitating early diagnosis and appropriate treatment.^[8]^

CDH results from a failure in the complete formation of the diaphragm. By the 8th week of gestation, the pleuroperitoneal membrane forms completely, with the right side developing before the left. Consequently, CDH can manifest as a left-sided posterolateral hernia, known as a Bochdalek hernia, which is much more common than a right-sided anteromedial hernia, referred to as a Morgagni hernia. While most CDHs, particularly large symptomatic hernial defects, are found during infancy, only 5% to 10% remain undetected until adulthood.^[13]^

A review article indicated that approximately 28% of patients with CDH were asymptomatic at initial presentation, while others exhibited a range of symptoms including bowel obstruction, pulmonary symptoms, pain, dysphagia, bleeding, and gastroesophageal reflux disease.^[7]^ Most patients had symptoms such as pain (37%) and pulmonary symptoms (36%), including dyspnea, cough, and shortness of breath related to Morgagni hernia, with complaints of obstruction occurring in 20% of cases.^[7]^ Our first patient experienced anterior chest tightness accompanied by intermittent right upper quadrant abdominal colic pain and chronic constipation, with symptom relief following surgery. The second patient experienced only intermittent fever without other specific associated symptoms related to CDH, which subsided after hernia reduction and repair. Most studies have reported pulmonary or gastrointestinal symptoms, with limited reports of fever alone as a primary or sole presentation.^[14]^

Diagnosis of CDH in adults relies on various modalities and clinical presentations. Chest radiography may reveal partial opacification of the hemithorax, elevation of the hemidiaphragm, blunting of the costophrenic angle, distortion of the diaphragm, curling of the gastric tube into the thorax, deviation of the mediastinum, or the presence of an air-filled bowel in the thoracic cavity.^[15]^ CT is the most accurate diagnostic modality for CDH in adults. It not only confirms the diagnosis but also provides detailed information about the defect and its contents.^[16]^ Precise CT imaging allows for the detection of the exact anatomical location of the defect, its contents, and other anatomical abnormalities such as malrotation which aids in planning the optimal surgical approach. One study found that approximately 38% of CDHs in adults are misdiagnosed as other conditions, such as pneumothorax, pleural effusion, empyema, or lung cysts, if a CT scan is not performed.^[17]^ Other diagnostic tools, such as esophagogastroduodenoscopy, ultrasound, and upper gastrointestinal contrast studies, are less commonly used.^[5]^ No other literature has described a similar diagnostic process involving a gallium scan as we did.

Surgery remains the primary treatment strategy for CDH in adults and can be accomplished using various approaches. For nontraumatic diaphragmatic hernias, surgical repair is generally recommended,^[18]^ except when general anesthesia is contraindicated. Given the risk of incarceration, all adults with CDHs should undergo surgical repair to prevent the significantly increased morbidity and mortality associated with emergency interventions.

Traditionally, laparotomy or thoracotomy has been the feasible approach for treating CDH in adults. With improvements in minimally invasive techniques, laparoscopic or thoracoscopic approaches are now considered alternatives that offer better postoperative recovery, shorter hospital stays, and reduced operative pain.^[19,20]^ A minimally invasive approach is recommended for stable patients, whereas laparotomy is used for unstable patients.^[18]^ Studies suggest that postoperative morbidity rates are comparable whether the surgery is performed using an open or minimally invasive technique.^[21]^

However, the rigidity of laparoscopic instruments, the limitation of 2-D imaging, lengthy procedures, and hand tremors pose challenges for surgeons. In recent decades, robot-assisted surgery has increasingly been applied to various abdominal procedures. With its improved dexterity, enhanced resolution, and stability of movements,^[22]^ robot-assisted surgery offers greater accuracy in sac excision, adhesion management, and minimizes injuries to incarcerated tissues or intestines.

We have observed difficulties associated with the reduction of Morgagni or Bochdalek hernias due to their anterior or posterior anatomical locations. Using a robotic platform, we can achieve better precision in hernia reduction.^[22]^ Structures commonly found in the Bochdalek hernia sac, such as the small bowel, colon, stomach, liver, spleen, omental tissues, or even the kidney,^[23,24]^ suggest that the robotic platform can provide a more efficient solution for surgeons facing these challenges. This approach may decrease postoperative complication rates and improve recovery. Studies have shown that robotic repair, compared to laparoscopic techniques, can result in shorter hospital stays.^[12]^

Some studies have highlighted the favorable postoperative outcomes of the transabdominal approach for CDH repair in adults.^[11,25,26]^ These studies suggest that the robotic platform can be considered an appropriate procedure for adult patients with CDH. However, other research indicates that the transthoracic approach may also be a suitable treatment for CDH in adults.^[27,28]^ Lambert et al reported a case of transabdominal-approach robotic surgery for multi-visceral procedures, including Morgagni hernia repair and low anterior resection for rectal cancer,^[29]^ highlighting the versatility of robotic platforms in handling complex surgeries.

After reducing the hernia and excising the hernia sacs, we used direct sutures to close the hernia defect, with or without mesh reinforcement. Available methods for managing CDHs in adults include primary suturing alone, primary suturing with mesh placement to strengthen the defect, or mesh placement without primary suturing for reconstructing diaphragmatic integrity.^[30]^ There is no definitive best solution for restoring integrity; therefore, the optimal approach should be determined based on individual patient factors.

## 4. Conclusion

We present 2 adult cases of robot-assisted hernia repair for CDHs. The rarity of CDHs in adults poses a significant challenge for clinical diagnosis. Our experience suggests that robotic surgery is a valuable treatment option for this condition. With the advantages of flexible joint movements and clear visualization provided by the robotic platform, we observed comparable safety and improved precision compared to laparoscopy. However, due to the sample size, we could not definitively verify the advantages of robotic versus laparoscopic approaches. For experienced surgeons, the robotic approach for diaphragmatic hernia repair can be considered a viable alternative to other minimally invasive techniques for selected patients. Further research is needed to more thoroughly evaluate robotic approaches for managing CDH in adults.

## 5. Limitations

This study has several limitations, including its small sample size, retrospective analysis, and single-center design. Due to the limited number of cases, we cannot clearly assess the differences in outcomes between robotic and laparoscopic surgeries; however, we anticipate that further studies will address this gap. Additionally, the retrospective analysis may have introduced treatment choice bias. The rarity of this population makes it challenging to conduct a randomized clinical trial.

## Author contributions

**Conceptualization:** Yu-Jen Huang, Yue-Lin Fang.

**Data curation:** Yu-Jen Huang.

**Formal analysis:** Yu-Jen Huang.

**Investigation:** Yu-Jen Huang.

**Methodology:** Yu-Jen Huang, Yue-Lin Fang.

**Project administration:** Yu-Jen Huang, Yue-Lin Fang.

**Supervision:** Yue-Lin Fang.

**Writing – original draft:** Yu-Jen Huang.

**Writing – review & editing:** Yu-Jen Huang, Yue-Lin Fang.
